# Harnessing the Power of Our Immune System: The Antimicrobial and Antibiofilm Properties of Nitric Oxide

**DOI:** 10.3390/microorganisms12122543

**Published:** 2024-12-10

**Authors:** Jonathan Matthew Roberts, Scarlet Milo, Daniel Gary Metcalf

**Affiliations:** Advanced Wound Care Research & Development, Convatec, Deeside Industrial Park, Deeside CH5 2NU, UK; jonathan.roberts@convatec.com (J.M.R.); scarlet.milo@convatec.com (S.M.)

**Keywords:** nitric oxide, wound, hard-to-heal, biofilm, antimicrobial

## Abstract

Nitric oxide (NO) is a free radical of the human innate immune response to invading pathogens. NO, produced by nitric oxide synthases (NOSs), is used by the immune system to kill microorganisms encapsulated within phagosomes via protein and DNA disruption. Owing to its ability to disperse biofilm-bound microorganisms, penetrate the biofilm matrix, and act as a signal molecule, NO may also be effective as an antibiofilm agent. NO can be considered an underappreciated antimicrobial that could be levied against infected, at-risk, and hard-to-heal wounds due to the inherent lack of bacterial resistance, and tolerance by human tissues. NO produced within a wound dressing may be an effective method of disrupting biofilms and killing microorganisms in hard-to-heal wounds such as diabetic foot ulcers, venous leg ulcers, and pressure injuries. We have conducted a narrative review of the evidence underlying the key antimicrobial and antibiofilm mechanisms of action of NO for it to serve as an exogenously-produced antimicrobial agent in dressings used in the treatment of hard-to-heal wounds.

## 1. Introduction

The human race’s precious resource of antibiotics able to effectively fight bacterial infections continues to decline. This is due, in part, to the inappropriate use and overuse of antibiotic therapy and consequent acquired and exchanged bacterial genetic resistance, alongside our emergent appreciation of biofilm tolerance in chronic infections [1,2]. Antibiotics generally target single bacterial survival mechanisms, rendering their actions susceptible to pressures of resistance, while standard antiseptics at safe levels cannot always fully penetrate protected biofilm communities [3]. When the human innate immune system is compromised in high-risk patient populations (such as the immunosuppressed, immobile or elderly, or those with diabetes or circulatory diseases), the challenge of sub-optimal antimicrobial treatments threatens the health and future prosperity of all.

Whilst the prospect of new armouries of antibiotic drugs appearing is unlikely, antibiofilm therapies are emerging that show promise in overcoming the combined challenge of antibiotic resistance and biofilm tolerance [4]. Examples include inhaled therapies for lung biofilm infections [5], antibiofilm wound and skin cleansers [6], and antibiofilm wound dressings, which have chemical or physical biofilm-disrupting actions to potentiate antiseptic agents [6,7]. A pragmatic and effective approach (similar to one widely used by the general population as daily oral hygiene) is the routine, repetitive combination of physical and chemical targeting of wound and skin biofilm, using cleansing, debridement, refashioning, and wound dressings containing antibiofilm and antiseptic agents [8,9]. 

Wound dressings incorporating ionic silver [10], and more recently with additional antibiofilm agents [9], have been used to improve healing outcomes of hard-to-heal wounds and have been reviewed elsewhere [11,12]. The microbicidal activity of silver is well-established and has proved a cornerstone of clinical management in the control of microbial bioburden and improved wound healing rates [13]. Nevertheless, an antimicrobial agent which combines multiple microbial targets (making resistance unlikely), inherent antibiofilm activity, and tolerance by human biological systems (i.e., is ‘natural’) that can be formulated into realisable treatment delivery vehicles would be an attractive proposition in tackling chronic biofilm infections, helping to preserve antibiotics for the highest risk situations. One approach would be to take inspiration from the human innate immune system, which has evolved multiple antimicrobial mechanisms as a first-line defence against invading pathogens [14]. These include the production of complex molecules such as antimicrobial peptides, cytokines, and antimicrobial enzymes and proteins such as lysozyme, neutrophil elastase [15], defensins, and lactoferrin [16]. Additionally, small molecule antimicrobial agents are generated by other neutrophil enzymes of the innate immune system, including hydrogen peroxide (H_2_O_2_) and superoxide (O_2_^−^) via superoxide dismutase and hypochlorous acid (HOCl) via myeloperoxidase, in the oxidative burst. Some of these antimicrobial agents have been synthesised and employed in liquid formulations (e.g., in H_2_O_2_-containing mouthwashes and HOCl-containing skin and wound cleansers) [17]. 

Another key product of the oxidative burst is nitric oxide (NO), which is produced by nitric oxide synthase (NOS) enzymes. NO is an attractive option as an exogenous antimicrobial and antibiofilm agent, as there is evolutionary proof of effectiveness against pathogens and tolerability. As a free radical, NO is relatively long-lasting yet is still quick to clear, and its reactions are reasonably specific, e.g., with metals and with radicals to generate other reactive nitrogen species (RNS). Following much interest in 1992’s ‘Molecule of the Year’ [18], there has been a resurgence of interest in NO utilisation, yet its potential in health care has been largely unrealised to date, most likely due to technical challenges in formulating effective, long-lasting treatments. Recently, technical advancements have been made in the use of NO in the treatment of infections, e.g., of the lung [19], diabetic foot ulcers [20], and burn wounds [21]. NO-based formulations currently marketed or undergoing clinical trials are listed in Table 1.

The aim of this narrative review is to summarise the key antimicrobial and antibiofilm properties of NO and to highlight the potential of this molecule as a novel solution to the global threat of antibiotic resistance and biofilm tolerance. Realisation of this may improve the lives of patients suffering from chronic infections, thus reducing their associated burden on healthcare systems. This work aims to outline a comprehensive overview of the mode of action of NO, illuminating its dual functionality as a potent antimicrobial agent and biofilm disruptor and highlighting its capabilities in combating chronic infections. This exploration is particularly relevant for advancing NO-based therapeutics in wound care, where persistent biofilms and resistant pathogens significantly hinder the healing pathway. A deeper understanding of NO’s action will pave the way for innovative, targeted treatments, addressing a pressing clinical need and enhancing patient outcomes. 

## 2. The Innate Nitric Oxide System

### 2.1. The Immune Response to Pathogens

**Figure 1:**
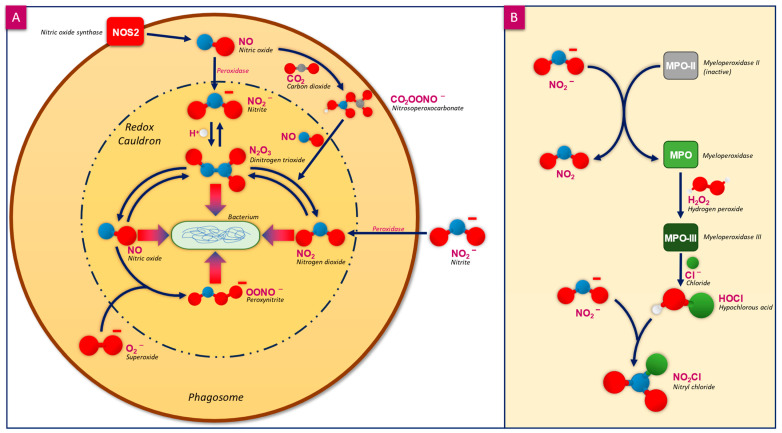
(**A**) Bacteria trapped within the phagosome of a phagocyte of our immune response experience a series of attacks from different RNS produced within the “redox cauldron” such as peroxynitrite [38]. NOS acts as a source of NO for the phagosome, which, due to the acidic environment, reacts to form different RNS. The reaction with carbon dioxide (CO_2_) forms nitrosoperoxocarbonate, which further reacts with NO to form either N_2_O_3_ or NO_2_. Peroxidase also plays a key role within the phagosome, catalysing the reaction of NO into NO_2_^−^ and then into NO_2,_ which interacts with the trapped bacterium. (Adapted and modified from Wink et al., 2011 [35]). (**B**) Free nitrite is used by myeloperoxidase in the generation of hypochlorous acid (HOCl) within neutrophils as part of the immune response, which produces NO_2_ as a byproduct. Nitrite further reacts with HOCl to produce the RNS nitryl chloride (NO_2_Cl), which, similar to OONO^−^, nitrates tyrosine within proteins [33,39].

Nitric oxide (NO) is a simple gaseous molecule generated as part of the innate immune response to pathogens (Figure 1) [26]. Owing to its small size, (30 Da), low polarity, and weak hydrophobicity [27,28,29] it can readily diffuse through tissues and body fluids, passing through cell membranes with little hindrance. NO possesses an unpaired electron, making it a free radical that is able to react with other free radicals and metals when under biological conditions [30]; however, it is relatively stable and does not react with itself. NO has a physiological half-life that ranges from seconds to minutes depending on its environment [29]. The formation of NO is catalysed by nitrogen synthases (NOSs) of the immune system, where L-arginine is oxidised in a two-step reaction into one molecule of NO and one molecule of L-citrulline, using 1.5 molecules of nicotinamide adenine dinucleotide phosphate (NADPH) and two molecules of oxygen (O_2_) [31]. The reaction rate is dependent on the concentration of L-arginine present, where a lack of L-arginine will decrease NO production [32]. NOSs are found in many immune system cells, such as macrophages and neutrophils, and are regulated by cytokines [26]. The activity of NO is not restricted to its locality of production as it can be transported over large distances in the form of S-nitrosothiols or S-nitrosylated proteins, which release NO spontaneously, or when cleaved by ectoenzymes found in T and B cells of the immune system [26]. NO and RNS may also be generated at sites of infection from the more stable circulating nitrite (NO_2_^−^) via the peroxidase pathway (Figure 1B) under acidic conditions, which allows immune cells lacking NOS to produce and use NO against pathogens [26,33]. 

NO is only one part of the innate immune response, a considerable proportion of which comes from other reactive nitrogen species (RNS) [34]. One key area where RNS and NO have activity is within the phagosome (Figure 1A), defined as a method of killing invading bacteria within a “redox cauldron” [35]. The phagosome is formed within neutrophils and is a vacuole formed around invading pathogens, creating a hostile environment [36,37] which has a low pH, facilitating the generation of reactive oxygen species (ROS) by NADPH oxidase (such as superoxide (O_2_^−^), and RNS from NO generated by inducible NOS from L-arginine). Within the phagosome, NO undergoes a series of reactions within the low pH environment, some of which are catalysed by peroxidase and others reacting directly with H^+^, producing various RNS including NO_2_^−^, N_2_O_3_ (nitrous anhydride), and OONO^−^ (peroxynitrite), which will interact with the captured bacteria [35,37]. The RNS species in existence are in constant flux, changing between NO, N_2_O_3_, and NO_2_, thus giving it the “redox cauldron” moniker [35]. As NO diffuses into the bacteria, it undergoes further reactions, producing additional RNS that have a limited ability to cross the lipid membrane due to their greater reactivity towards lipids, therefore causing damage to intracellular components of the bacteria. The resultant death of the captured bacteria is induced by the multiple mechanisms of action of the toxic RNS [27]. 

### 2.2. Mammalian Tolerance to NO and Other RNS

The exposure of mammalian cells to RNS has both positive and negative implications. NO has been shown to have minimal cytotoxicity across a variety of mammalian cell structures whilst also possessing a protective role against the toxicity of agents such as ROS [40,41]. The toxic effects of NO against mammalian cells are lessened compared with its effects against bacteria, owing to the inherent protection mechanisms. One such method of protection is through tight regulation of NO production by restricting substrates (such as L-arginine), allowing levels of NO to remain within cellular tolerance [42]. Levels of NO can also be reduced by switching off NOS through post-translational changes, further reducing the production of NO [43]. Since NO diffuses easily and rapidly, it cannot easily be contained for reuptake, so it must be removed through a series of reactions [44,45]. NO is removed from tissues via rapid reaction with oxyhaemoglobin and oxymyoglobin in the blood, which forms the non-damaging end product, NO_2_^−^. Large concentrations of NO can be depleted by the high concentration of haemoglobin in the blood, where it is drawn out of the tissues and prevented from re-entering the cells. This process of removal by blood takes mere seconds in vivo [45]. Therefore, damage to mammalian cells is dose-dependent. Indeed, the delivered concentration and generation profile of NO in future products should be taken into consideration [46,47]; maintaining levels that kill bacteria whilst remaining tolerable to mammalian cells is a fundamental aspect of the innate immune system.

## 3. Antimicrobial Action

### 3.1. Penetration of the Cell Wall

**Figure 2:**
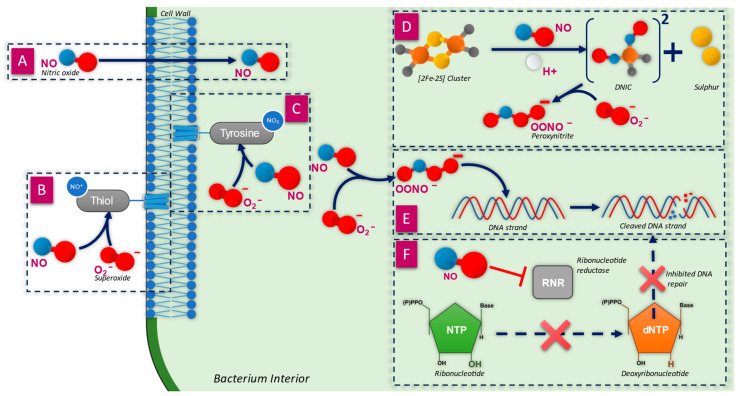
A summary of the broad-spectrum antimicrobial mechanisms of action of NO and RNS within a bacterium cell. (**A**) NO freely diffuses through the cell membrane due to its small size and low charge [28]. (**B**) NO reacts with sulphur-containing thiol groups on outer cell wall-embedded proteins, deactivating them [48]. (**C**) NO deactivates proteins embedded within the bacteria on the cell wall, targeting sulphur-containing tyrosine groups to deactivate them [48]. (**D**) The iron–sulphur-containing proteins are one of the primary targets of NO. The reaction with NO, facilitated by H+, produces two dinitrosyl iron complexes (DNICs) molecules and two free sulphur molecules. The DNICs can further react with oxygen to form OONO^−^ [49]. (**E**) Peroxynitrite reacts with DNA strands, resulting in deamination and oxidative damage, which will eventually lead to DNA cleavage. Without a functional RNR enzyme producing more dNTP molecules, the damaged DNA is no longer repaired or replaced [50]. (**F**) Ribonucleotide reductase (RNR) is a catalyst for the conversion of nucleotides (NTP) into deoxynucleotides (dNTP), which are used for DNA repair and replication. NO has strong reactivity towards the tyrosyl free radicals within RNR, which changes the structure of the enzyme, inhibiting it [51].

NO is a gaseous molecule that is able to freely diffuse across the cellular membranes (Figure 2A) of both bacterial and mammalian cells due to its small stokes radius, low polarity, and weak hydrophobicity, in a fashion similar to O_2_ [27,28]. 

The rate of diffusion into bacteria is influenced by the structure of the cell membrane, as observed for Gram-positive bacteria (such as *Staphylococcus aureus*), which require higher doses of NO for eradication due to the thick peptidoglycan layer which surrounds the cell membrane [52]. Gram-negative bacteria, such as *Pseudomonas aeruginosa*, are more susceptible to NO as they lack a peptidoglycan layer and have a lipid-rich outer membrane [53]. Unlike NO, other RNS, such as OONO^−^, cannot easily diffuse through the cell wall owing to their ionic charge [35] but can do so when they lose the charge. For example, OONO^−^ becomes peroxynitrous acid (HNO_3_), which can diffuse across the membrane [27]. The requirement for RNS to diffuse is less significant than it is for NO, as RNS may be formed in situ within bacterial cells by NO and O_2_^−^ [27]. 

### 3.2. Inactivation of Membrane Proteins

The bacterial cell wall contains an array of membrane proteins which enable interaction between the bacteria and its environment in processes such as pathogenicity, signal transduction, nutrient exchange, and protection from oxidative stress [54,55]. The proteins found within bacterial cell membranes make up around 20–30% of all proteins within the cell, as derived from genomic DNA sequencing [55,56]. The structure of these proteins is either α-helical or β-barrel, with a greater abundance of β-barrel in outer membranes and α-helices being more abundant in the inner membrane. Membrane proteins of significance include OmpF and OmpC [56] (two porins attributed to silver resistance [57]) and sucrose porins (used to transport sugars across the membrane [58]). 

The function of many outer membrane proteins is enabled by sulfhydryl or thiol groups (Figure 2B), particularly for active transport and oxidative phosphorylation [59]. Analysis of plasma membranes of *Streptomyces albus* and *Escherichia coli* found 5.2 and 3.4 mol/50 kg protein of thiol groups, respectively, verifying the importance of these groups in the structural organisation of membrane proteins [60]. The modification of thiol groups within outer membrane proteins has been identified as a target for bacteriostatic and antibiotic action [61]. It has been shown that the RNS dinitrogen trioxide (N_2_O_3_) reacts with thiol groups of outer membrane proteins, resulting in nitrosation of amines [48], which is known as S-nitrosylation (the addition of a NO^+^ to a donor group, in this case, a thiol group) [30]. S-nitrosylation within proteins results in conformational changes, particularly if two neighbouring thiol-containing cysteine residues are nitrosylated, resulting in the inhibition of di-sulphide bridge formation. Subsequent protein modifications may inhibit the function of the protein, alter interactions with other proteins, or cause protein aggregation [61]. 

There are a group of proteins found embedded within the cytoplasm facing the cell membrane which contain tyrosine groups (Figure 2C). These are crucial to a number of cellular processes, including bacterial pathogenicity and biofilm formation, by acting as a control for different pathways through tyrosine phosphorylation [62]. However, the tyrosine group within these proteins is susceptible to nitration by OONO^−^, thus changing the structure and function of the protein. The nitration reaction is chemically distinct from S-nitrosylation; nitration is the addition of a NO_2_ group, whereas nitrosylation is the addition of NO^+^ [30]. The nitration of tyrosine results in the formation of a tyrosyl radical, which will further react to form 3-nitrotyrosine, a known biomarker for nitrosative stress [61]. Therefore, changes to the function of tyrosine-containing proteins may reduce the overall pathogenicity of the bacteria and biofilm formation by impairing their ability to be phosphorylated and dephosphorylated as part of quorum sensing pathways [62]. 

### 3.3. Breakdown of the Cell Wall

A study by Deupree and Schoenfisch (2009) found that exposure of *E. coli* and *P. aeruginosa* to NO resulted in membrane degradation, debris formation around the cell surface, cellular collapse, and lysis of the cell. It was theorised that OONO^−^ at the cell membrane reacts with lipids via radical peroxidation, resulting in the breakdown of the membrane structure [63]. The oxidation reactions are dose dependent, as NO can both induce and inhibit the reaction, with higher doses reducing lipid oxidation [64]. It was observed that higher levels of NO over short periods of time are more damaging to Gram-negative bacteria than a sustained low-level dose [63]. Further disruption to the cell membrane occurs when components, such as the aforementioned proteins, are damaged, increasing membrane permeability. This has been observed when combining NO and antibiotics, leading to an increased bacterial susceptibility to antibiotics, which slows the development of antibiotic resistance [65].

### 3.4. Inactivation of Iron–Sulphur-Containing Proteins

The primary targets for NO are metal-containing proteins; however, the products of its reaction with dioxygen (NO_x_), superoxide (ONOO^−^), and transition metals (NO^+^, M-NO) are observed to react with nucleic acids, amino acid side chains, and a variety of low molecular weight amines and thiols [66]. Iron–sulphur (Fe-S) clusters are theorised to be one of the earliest to evolve iron cofactors in biology [67,68], from a time when the environment was anaerobic during the first billion years of life, and Fe-S clusters enabled electron transfer for ancient proteins [69]. Fe-S clusters are found in many different protein assemblies, the most common being [2Fe-2S] and [4Fe-4S], usually found in cysteine residues [70]. The assemblies are generally stable at different oxidation states and have physiologically relevant redox potential characteristics, meaning that Fe-S clusters may act as environmental sensors by reacting to cellular oxidants and changing the states of other molecules [71]. The role of Fe-S clusters is primarily electron transfer, although Fe-S cluster-containing proteins also have other roles in pathways, such as nitrogen fixation and gene regulation, dictating transcriptional and translational rates [67,72]. An example of key Fe-S cluster-containing proteins is fumarate and nitrate reductase (FNR) found in *E. coli*. This Fe-S-containing protein is a global regulator of over 100 genes where the binding to DNA is enabled by a [4Fe-4S]^2+^ cluster [71]; therefore, inactivating this will impact many different cellular processes. Aconitases found in *E. coli* and *Bacillus subtilis* are also important Fe-S-containing proteins and are involved in translational regulation [73,74], enabled by a [4Fe-4S] cluster which helps it to bind to RNA, stabilising or blocking its translation. Disrupting the [4Fe-4S] cluster, therefore, will disrupt its ability to bind to RNA. Fe-S clusters are highly reactive with NO (Figure 2D), resulting in modifications and damage to proteins [35,49]. The reaction with NO produces dinitrosyl iron complexes (DNICs), as NO binds to the iron atoms, changing the shape of the cluster [66]. The formation of DNICs is used as a biomarker for NO toxicity [49]. NO has been shown to deactivate aconitase (an enzyme that catalyses the conversion of citrate to isocitrate in the tricarboxylic acid cycle) via reactions with the Fe-S cluster, thus reducing its ability to regulate translational functions [50]. Previously, attention has focused on the reaction of NO and ONOO^−^ with the iron regulatory protein (IRP1) and related mitochondrial aconitases. IRP1 contains a 4Fe-4S cluster and exhibits aconitase activity. The cluster can undergo oxidation to an inactive 3Fe-4S form and can be completely degraded to apo-IRP1. Apo-IRP1 plays a role in iron homeostasis by binding to iron regulatory elements (IREs) contained in the 3′- and 5′-untranslated regions of some mRNAs. Via this mechanism, IRP1 can inhibit the translation of the iron storage protein ferritin but activate the translation of the transferrin receptor protein. Nitric oxide provides a plausible mechanism for the conversion of IRP1 from its Fe-S cluster-containing form to the RNA-binding form. Indeed, it has been shown that the reaction of NO, both with the IRP1 and mitochondrial aconitase, results in the formation of protein-bound DNICs [75]. FNR is also inactivated by NO via rapid conversion of its [4Fe-4S]^2+^ cluster to [2Fe–2S]^2+^, which induces a conformational change to the enzyme [72]. A study by Ren et al. (2008) found that exposure of *E. coli* to NO resulted in inhibition of its growth owing to modifications to Fe-S cluster-containing proteins. It was shown that Fe-S cluster-containing dihydroxyacid dehydratase (an essential enzyme for branched-chain amino acid biosynthesis) formed a DNIC-bound species, thus inactivating it, further highlighting the key role that Fe-S clusters play in bacterial survival [76].

The formation of DNICs is not the only method of attack on bacteria, but the creation thereof can also release free iron ions into the cell, where they bind to DNA. Iron binding to DNA results in cleavage through oxidation reactions [35,77]. Further damage may be caused to the bacteria as DNICs react with O_2_ to form OONO^−^, thereby inducing nitration of tyrosine-containing proteins, changing their structure and function [49,78]. 

### 3.5. Direct Damage to DNA Strands

As RNS diffuse through the bacterial cell, they begin to interact with plasmid DNA strands (Figure 2E), which has been shown to induce mutagenicity [79]. There are two primary methods of DNA damage, each induced by either OONO^−^ or N_2_O_3_. Firstly, the damage caused to DNA by OONO^−^ is more efficient than NO alone, resulting in single-strand fragmentation, and has been shown to occur at concentrations as low as 2–5 µM. OONO^−^ reacts directly with the sugar moiety of the sugar-phosphate backbone, likely due to hydrogen abstraction, forming a sugar radical which undergoes subsequent reactions [50,80]. Secondly, N_2_O_3_ directly attacks DNA, leading to deamination through the formation of a diazonium ion. This undergoes further hydrolysis, which completes the deamination. This process changes stable DNA bases into unstable bases, for example, adenine into hypoxanthine and guanine into xanthine. The modification of bases leads to mispairing, e.g., the formation of xanthine causes a G:C base pairing to become A:T [50]. The instability of xanthine forms a basic site within the DNA, which may result in cleavage by endonucleases to form single-strand breaks [81].

### 3.6. Inhibition of DNA Synthesis and Repair

Ribonucleotide reductase (RNR) is a key enzyme for the synthesis and repair of DNA strands, required for the conversion of ribonucleotides into deoxyribonucleotides (Figure 2F), the building blocks of DNA [51,82]. The amount of available deoxyribonucleotides within the cell is essential for the stability of the genome and is carefully balanced by RNR activity [83]. NO has a strong affinity towards the amino groups of the RNR enzyme, modifying the structure and ultimately inhibiting its function. A study by Lepoivre et al. (1991) found NO has strong reactivity with an iron-containing tyrosyl residue within RNR, which provides a structural bridge within, resulting in a change to the overall structure and switches the enzyme off [51]. There may also be reactions of NO with cysteine groups within RNR by nitrosation, further inhibiting the enzyme function [84]. The inhibition of RNR, therefore, prevents the reduction of hydroxyls (OH) of ribonucleotides to a [H] atom to form deoxyribonucleotides [51,84]. 

DNA alkylation, where an alkyl group is added to a DNA base, constitutes major damage to a cell as it is both potentially cytotoxic and mutagenic [85]. Some bacteria possess a class of enzymes called *O^6^*-alkylguanine-DNA-alkyltransferases (AGTs), which protect DNA from mutations caused by alkylating agents, particularly alkylated guanines on the *O^6^* position [86]. AGTs can repair the damage as they contain a thiol-containing cysteine group at its active site, which displaces *O^6^*-alkyl groups from guanine residues [82,87]. It has been shown that AGTs are sensitive to disruption by NO, which reacts with the thiol group within the active site, switching the enzyme off [87]. This process is generally irreversible as the degradation of the protein is rapid upon formation of the sulphur-NO adduct, which leads to a permanent loss of DNA repairability for the cell [87,88].

## 4. Antibiofilm Action

### 4.1. Biofilm Dispersal—c-di-GMP Pathway

**Figure 3:**
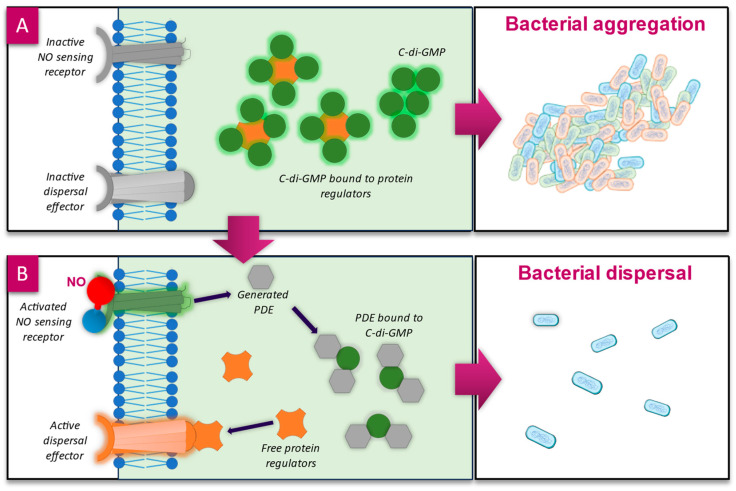
(**A**) Cyclic-diguanylate-guanosine monophosphate (c-di-GMP) within the bacterial cell regulates biofilm aggregation of bacteria. As the concentration of c-di-GMP increases, biofilm formation increases. It is thought that c-di-GMP binds to protein regulators of the dispersal proteins (such as proteins for flagellum movement), inactivating them. (**B**) NO binds to the haem groups of NO sensing receptor proteins [97] on the outside of the cell, activating them; this induces an upregulation of phosphodiesterase (PDE). PDE binds to and degrades c-di-GMP-releasing protein regulators; the resulting signal cascade ends with the activation of the dispersal proteins [103,104].

It is estimated that up to 80% of prokaryotes live in a biofilm state, ref. [89] and that biofilms account for 65% of microbial infections, as well as 78.2% of chronic infections in humans [90,91]. The formation of a biofilm follows a sequential process: free-floating planktonic bacteria and fungi express changes to their genes to become adherent to surfaces or each other, attached microorganisms form microcolonies encased in self-produced and acquired extracellular polymeric substances (EPSs), and the biofilm matures and may eventually disperse to spread colonies elsewhere [92]. The formation of EPSs is key to the survivability of microorganisms within as it provides an environment that promotes cohesion, adhesion, maintenance of optimum environmental conditions (such as pH and nutrient availability), protection from antimicrobial agents (e.g., antiseptics, antibiotics, and the immune system), and enables communication via quorum sensing between bacteria [89]. The promotion of bacteria from a planktonic state into a biofilm state, and vice versa, is controlled by the secondary messenger molecule cyclic diguanylate monophosphate (c-di-GMP), which influences bacterial behaviour such as surface adhesion and adaption, motility, and virulence [93,94]. Since most of the downstream targets of c-di-GMP are involved in EPS formation and maintenance [95], biofilm formation increases as the concentration of c-di-GMP within the cells increases (Figure 3A), and as levels decrease, bacteria return to a planktonic state [96]. 

The activity of c-di-GMP is understood to be regulated by endogenously produced NO during the late stages of biofilm development as a means to induce biofilm dispersal. NO acts as a signal molecule and binds to NO sensing receptor proteins at the cell wall, which in *P. aeruginosa* is the chemotaxis transducer protein biofilm dispersion locus A (BdlA), and in other bacteria is the haem-nitric oxide/oxygen protein (H-NOX). Both of these proteins contain haem groups, for which NO has a high affinity, inducing activation of the protein and subsequent stimulation of phosphodiesterase (PDE) activity [97]. PDE interacts with and degrades c-di-GMP, reducing its concentration within the cell (Figure 3B), resulting in the activation of protein regulators, which promote the planktonic phenotype [48,98]. This process of NO-mediated dispersal is conserved across different species, including *P. aeruginosa*, *S. aureus*, *E. coli*, *Fusobacterium nucleatum*, and *Vibrio cholerae* [95,99]. 

A study by Barraud et al. (2009) observed an average reduction of total biofilms of 63% across different species when exposed to NO at picomolar and low nanomolar concentrations, levels that are safe for human cells [100]. It has been shown, therefore, that NO can be exogenously added to biofilms to artificially induce dispersal, as has also been shown in studies using NO donor prodrugs targeting *P. aeruginosa* and *E. coli* biofilms [101,102]. The dispersal effect of NO can be further enhanced when combined with tobramycin, and a combination with colistin mostly eradicating biofilms [93]. From this research, we can surmise that a NO-generating wound dressing, either alone or in combination with other antimicrobials, would be a promising method of biofilm control, particularly in hard-to-heal wounds which are known to harbour recalcitrant biofilms (Malone et al., 2017) [91] and require advanced antibiofilm dressings [7] and multi-step biofilm-targeting treatment protocols [8,9]. 

### 4.2. Influencing Quorum Sensing Pathways

Quorum sensing (QS) refers to the process by which bacteria communicate via a ‘chemical vocabulary’. Gene expression is regulated in response to small molecules known as autoinducers (typically acylated homoserine lactone molecules in Gram-negative bacteria or oligopeptides in Gram-positive bacteria), which are synthesised within the cell and freely diffuse across the cell membrane. As cell density increases, so does the concentration of autoinducers in the surrounding environment. When the autoinducer concentration passes a threshold level, these molecules bind to their respective receptor and initiate a signal transduction cascade downstream, ultimately resulting in the repression or expression of specific genes [105]. QS has been shown to mediate several bacterial processes, including bioluminescence, sporulation, competence, antibiotic production, biofilm formation and production of virulence factors [106].

As previously discussed, NO is toxic to bacteria at high concentrations (approximately micromolar), outlining one line of defence that NO-producing eukaryotes have against bacterial infection. Bacteria may also encounter relatively high concentrations of NO during denitrification (a process by which some bacteria can respire on nitrate or nitrite under oxygen-limiting conditions). NO is subsequently reduced to nitrous oxide by NO reductase. The expression of both nitrite and NO reductase in *P. aeruginosa* biofilms was found to be QS-dependent, suggesting that QS is responsible for the maintenance of NO levels in this organism [107]. 

Recent studies have reported the response of bacteria to relatively low, nontoxic concentrations of NO (approximately nanomolar to micromolar) to elicit physiological responses other than those involved in denitrification and detoxification (i.e., other than processes primarily aimed at the elimination of NO from the cell). The biofilm-related responses to NO are known to be distinctly concentration-dependent [95], the most extensively studied example of which is in *P. aeruginosa*. In an early study of the effect of NO on biofilm formation, it was shown that *P. aeruginosa* remains in the biofilm state until exposed to 25 nM to 2.5 mM of sodium nitroprusside (SNP, an NO donor), which corresponds to approximately 0.025–2500 nM of NO, at which point they revert to a free-swimming, planktonic lifestyle. When the bacteria are exposed to NO concentrations greater than ~25 μM, however, biofilm formation was enhanced relative to biofilm formation in the absence of NO [103].

Nevertheless, NO has been shown to be effective against both Gram-negative and Gram-positive biofilm bacteria [108]. As such, researchers have been investigating applications of NO as an antibiofilm agent for therapeutic applications (Table 2).

The consistent delivery of NO gas to the targeted therapeutic location remains a significant challenge in harnessing this technology. Thus, NO-releasing materials are being explored as antibiofilm agents for the treatment of pathogens.

N-diazeniumdiolates (NONOates) have been investigated as NO donors by Schoenfisch et al. (2014). Owing to their instability at room temperature and in neutral or acidic solutions, they have been incorporated into macromolecule vehicles to deliver NO to bacteria for antibacterial and antibiofilm applications. These studies reported that at a 1–10 mg/mL dosage, surviving cells are suppressed to <0.001% after modification of silica nanoparticles, amphiphilic poly(amidoamine) dendrimers, and chitosan oligosaccharides with NONOates [112,113,114]. 

Whilst NO-releasing macromolecules are adequate for broad-spectrum bactericidal purposes, it has been theorised that a biofilm-specific NO prodrug may be more efficient. Barraud et al. (2012) synthesised the NO prodrug cephalosporin 3′-diazeniumdiolate, to investigate the release of NO by biofilm-specific β-lactamases. This compound was shown to display a dose-dependent effect on *P. aeruginosa* biofilm dispersal at a prodrug concentration of 2–200 µM [102]. 

Alternatives to NONOate-based NO-releasing compounds are also being investigated for antibiofilm purposes. Stable free radical nitroxides (RNO•) have been shown to mimic the biofilm dispersal effect of NO on *P. aeruginosa*. Sulemankhil et al. (2012) [110] have tested the bactericidal effect of enzyme-catalysed NO-releasing dressings on the pathogenic bacteria *Acinetobacter baumannii*, MRSA, and *P. aeruginosa*. A dressing releasing >200 ppm of NO (~7 mM NO) was found to induce a 1000-fold reduction in bacterial viability after 3 h [115]. Antibacterial potency may be enhanced by combining a NO-releasing drug with a traditional antibiotic. For example, in studies in which the QS inhibitor Fimbrolide was functionalised with a NO-releasing group and applied to *P. aeruginosa*, reduced production of virulence factors and less biofilm aggregation was observed than in studies with Fimbrolide alone [116].

NO also reduces the virulence of bacteria by interfering with QS pathways, as shown in *S. aureus*. Here, autoinducing peptides (AIPs) act as signal molecules and are detected by AgrC, which transfers a phosphate group to the enzyme AgrA. Activated AgrA then initiates the transcription of RNA containing the *agr* operon, resulting in increased virulence of *S. aureus*, which increases as cell density increases. NO is able to react with AgrA at multiple sites, resulting in its inhibition, thus preventing the transcription of the *agr* operon and reducing the overall virulence of the bacteria [105]. 

### 4.3. Interactions with Extracellular Polymeric Substances

Bacteria produce and acquire EPS during biofilm maturation, which acts as a means to control the environment to favour microbial proliferation and survival [89]. EPS is highly hydrated, containing up to 97% water [117], and accounts for between 50 and 90% of the organic matter of the overall biofilm structure [118]. The composition of EPS is influenced by environmental factors such as levels of oxygen and nitrogen, temperature, pH, nutrient availability, and also the surface to which the microorganisms are bound [119]. Polysaccharides constitute the majority of the organic matter within the EPS, but they also contain proteins, extracellular DNA, lipids, and other small molecules, some of which are neutrally charged and others that are polyanionic. The overall charge of the biofilm is influenced by the microorganisms within it. For example, Gram-negative bacteria produce biofilms containing uronic acids and ketal-linked pyruvates, resulting in an anionic charge. These charges within the EPS allow association with divalent cations such as calcium (Ca^2+^) and magnesium (Mg^2+^), which cross-link polymer strands to increase the binding forces of the biofilm [118]. These charges give the biofilm protection to charged antimicrobials and antibiotics (for example, the polyanionic exopolysaccharide alginate gives protection to pseudomonal biofilms from aminoglycosides, and the polysaccharides, Pel and Psl, give protection against tobramycin and gentamicin), preventing penetration to the microorganisms within [120] and contributing to increased tolerance of 10–1000 times that of planktonic bacteria [121].

An advantageous antibiofilm action of NO over charged or larger antimicrobial agents is the ability to easily penetrate through biofilms to reach microorganisms within [122]. This is not only due to the gaseous nature of the molecule (allowing it to easily diffuse through) but also due to its water solubility [123], small size [27], low polarity, and net neutral charge [124]. NO has a molecular weight of 30.01 g/mol [125], which is similar to O_2_, 31.99 g/mol [126], and considerably smaller than that of other antimicrobials such as molecular iodine, 253.809 g/mol [127], and polyhexamethylene biguanide (PHMB) which ranges between 500 and 6000 g/mol [128]. As NO passes through the biofilm, it may react with O_2_ to form RNS such as OONO^−^, which can induce nitrosative stresses and destabilise the matrix as it begins to accumulate, as shown in *P. aeruginosa* and *S. aureus* biofilms [103,129].

Accumulation of RNS within biofilms may further destabilise the matrix via interactions with polysaccharides and extracellular DNA (eDNA). It has been shown in vitro that RNS are able to depolymerise polysaccharides, causing the cleavage of the backbone and subsequent decrease in the size of the polysaccharides, in a process that is dependent on concentration and distance to the RNS generation site [130]. One target of NO and its intermediate nitrosonium cation (NO^+^) are glycosaminoglycans (GAGs) (mucopolysaccharides with roles in bacterial and fungal adhesion and infection of host tissues), which are expressed on the bacterial cell surface within the biofilm matrix, and within the cell itself [131]. In an acidic environment (~pH 4), NO^+^ nitrosates amino groups of GAGs, resulting in a structural change and potential cleavage of the glycosidic bonds within [131,132]. This may lead to a reduction in the adhesive and infective capability of bacteria and fungi. Other polysaccharides degraded and cleaved by NO include capsular polysaccharide SP1 in *Streptococcus pneumoniae* (a polysaccharide that increases tolerance to immune responses [133]), as well as alginates, Psl, and Pel (three polysaccharides which provide structure to the biofilm matrix within pseudomonal biofilms [134,135]). Disruption of polysaccharides, the main polymeric component of biofilms, leads to reduced biofilm viscoelasticity [136], potentially rendering the component microorganisms exposed and susceptible to antimicrobial agents.

In *P. aeruginosa* biofilms, Psl and eDNA combine to form eDNA-Psl fibres to increase the strength of the biofilm matrix [137]. This structure may be weakened, not only by the damage to Psl but also to eDNA by RNS. As previously discussed, OONO^−^ can cleave DNA [49]; therefore, it may exhibit similar actions against structural biofilm eDNA. This was recently observed in a study where *P. aeruginosa* biofilms were exposed to NO-releasing chitosan oligosaccharide scaffolds, where eDNA within the EPS matrix was visibly altered and degraded as visualised using fluorescence microscopy [136].

The RNS nitrous acid (HNO_2_), which breaks down into NO and NO_2_ under acidic conditions [138], has recently been shown to interact with the components of the EPS. A study by Chislett et al. (2021) found that exposure of pseudomonal biofilms to HNO_2_ resulted in its breakdown and detachment owing to disruption of the EPS. HNO_2_ reacts with ethers, esters, and peptide bonds, key intermolecular structural bonds within the EPS. It can also interact with amide bonds in large folded proteins, resulting in their breakdown via deaminative depolymerisation [139]. Therefore, disruption of polysaccharide, eDNA, and protein components of the biofilm EPS matrix by RNS leads to biofilm destabilisation and susceptibility.

## 5. Conclusions and Future Directions

The ongoing patient and economic burden of chronic infections, such as hard-to-heal wounds that are compromised by biofilms or local infection, suggests that there is still a need for additional antimicrobial technologies beyond traditional antiseptics and antibiotics. This need is due to myriad factors, including increasing bacterial resistance to antibiotics, the emergence and acceptance of biofilm tolerance to standard antimicrobials [1,91], reluctance around the perceived safety or efficacy of some traditional antimicrobials, and the challenge of formulating safe and effective antiseptic chemistry with effective dressing materials [6,8]. Advances in antibiofilm formulations have been made in recent years, including combinations of traditional antiseptics with additional antibiofilm agents, for example, PHMB with a surfactant [6] and ionic silver with a chelator and surfactant [7]. However, an all-in-one antimicrobial and antibiofilm agent, based on chemistry that is unlikely to be compromised by pressures of resistance, which can be formulated into beneficial wound dressings, has been elusive.

NO is emerging as a tangible option due to its multiple antimicrobial and antibiofilm actions [140]. As outlined herein, NO is generated rapidly in the phagosome in response to pathogens, acting on multiple cell wall proteins and enzymes and nucleic acid structural and process targets in microbes, while mammalian cells have evolved to tolerate such effects. Furthermore, NO has inherent antibiofilm properties, including the ability to trigger biofilm dispersal, interfere with biofilm quorum sensing communication, and direct action on biofilm EPS structural components. NO technology appears to be realisable by mimicking the “redox cauldron” employed by our immune system phagocytes (Figure 1), where NO can be generated on demand by the acidification of NO_2_^−^ within advanced dressing materials to provide antibiofilm action in vitro [141] and facilitate wound healing [20]. This emerging evidence for a next-generation, differentiated antimicrobial and antibiofilm technology will need to be supported by comparative laboratory, clinical and health-economic data before adoption by healthcare professionals and institutions in the ongoing battle against hard-to-heal wounds and, more widely, against antibiotic resistance. Future studies should focus on the comparative efficacy of NO with ‘traditional’ antimicrobial agents, such as ionic silver or PHMB, although it is important to note the challenges of this type of direct quantitative comparison, owing to fundamental differences in mechanisms of action between NO and traditional antimicrobials. For instance, NO operates through multiple pathways, including nitrosative and oxidative stress, disruption of iron–sulphur clusters, and interference with bacterial signalling pathways. These distinct mechanisms make it challenging to establish direct equivalence in terms of efficacy metrics. Nevertheless, exploring such comparisons in future research would provide valuable insights into the relative strengths and limitations of NO-based therapeutics. Furthermore, future comparisons must address key challenges, including the standardisation of experimental conditions, varying microbial susceptibilities, and differences in delivery mechanisms. Assessing the antimicrobial performance of NO in clinically relevant wound environments will be critical to translating laboratory findings into practical applications. Such comparative studies are imperative to establish NO as a viable and effective option in modern antimicrobial strategies. 

## Figures and Tables

**Table 1:** Nitric oxide formulations for human wound and skin care currently marketed or in clinical trials.

Product/Prototype	NO Technology	Status	Reference
EDX 110	Acidified nitrite in two-part superabsorbent wound dressing	Prototype wound dressing reported in the ProNOx1 clinical study in DFUs	[20]
N1O1 Nitric Oxide Activating Serum with Antioxidants	Acidified nitrite in a two-part aqueous formulation	Available over-the-counter (OTC)	[22]
NoWonder™ Foot Cleanser	Nitric oxide in two-part aqueous solution footbath	Described as available in the US. Clinical proof of concept completed in a 40-patient DFU study	[23]
NOX 1416	Acidified nitrite in a surfactant-containing aqueous foam formulation delivered in a two-part pump dispenser	Prototype wound care product subject to ongoing clinical registry [24]	[25]

**Table 2:** The effect of nitric oxide on bacterial biofilms. * indicates the concentration of NO released by SNP calculated according to the measurements in Barraud et al., 2009 [100].

Species	NO Source	NO Donor Concentration	Approximate NO Concentration	Effect on Biofilm	Reference
Gram-Negative					
*Pseudomonas aeruginosa*	Sodium nitroprusside (SNP)	25 nM–2.5 mM	0.025–2500 nM *	Reduction	[103]
*Pseudomonas aeruginosa*	SNP	>25 mM	>25,000 nM *	Reduction	[103]
*Serratia marcescens*	SNP	25–500 nM	0.025–0.5 nM *	Reduction	[103]
*Escherichia coli*	SNP	500 nM	0.5 nM *	Reduction	[100]
*Escherichia coli*	N-diazeniumdiolate (NONOate)	100 μM	~100–300 nM	Reduction	[109]
*Acinetobacter baumannii*	Gaseous NO	200 ppm	~7 mM	Reduction	[110]
Gram-Positive					
*Staphylococcus epidermidis*	SNP	10 μM	10 nM *	Reduction	[100]
*Staphylococcus aureus*	Gaseous NO	200 ppm	~7 mM	Reduction	[110]
*Staphylococcus aureus*	NONOate	1–1000 μM	>0.125 mM	Reduction	[111]
*Staphylococcus aureus*	NONOate	1–1000 μM	~900–2000 nM	Reduction	[111]
